# Evaluating Community Engagement Strategies to Manage Stigma in Two African Genomics Studies Involving People Living with Schizophrenia or Rheumatic Heart Disease

**DOI:** 10.1155/2021/9926495

**Published:** 2021-06-26

**Authors:** Megan M. Campbell, Olivia P. Matshabane, Sibonile Mqulwana, Michael Mndini, Mohamed Nagdee, Dan J. Stein, Jantina De Vries

**Affiliations:** ^1^Psychology Department, Rhodes University, Grahamstown, South Africa; ^2^Department of Medicine, University of Cape Town, Cape Town, South Africa; ^3^Department of Psychiatry & Neuroscience Institute, University of Cape Town, Cape Town, South Africa; ^4^Psychology Department, Fort England Hospital, Grahamstown, South Africa; ^5^SA MRC Unit on Risk & Resilience in Mental Disorders, Department of Psychiatry & Neuroscience Institute, University of Cape Town, Cape Town, South Africa

## Abstract

In global health research and genomics research specifically, community engagement has gained prominence in enhancing ethical conduct, particularly in managing the risk of stigmatization, but there is minimal scientific evidence on how to do this effectively. This article reports on community engagement evaluation strategies in two African genomics studies: the Stigma in African Genomics Research study and the Genomics of Schizophrenia in South African Xhosa People (SAX) study. Within the Stigma in African Genomics Research study, a self-report rating scale and open-ended questions were used to track participant responses to an experiential theatre workshop. The workshop focused on participant experiences of living with schizophrenia or rheumatic heart disease (RHD). While the schizophrenia group reported more alienation and less stigma resistance than the RHD group, both groups demonstrated increased stigma resistance over time, after participating in the workshops. Hearing from others living with and managing the same illness normalised participants' own experiences and encouraged them. Within the SAX study, a short rating scale and qualitative feedback methods were used to evaluate a Mental Health Literacy Day targeting mental health stigma. Information talks about (i) the symptoms of schizophrenia and treatment options and (ii) the illness experiences of a patient in recovery were rated as the most helpful on the day. Audience members reported that these talks challenged negative perceptions about severe mental illness. Three important learnings emerged from these evaluations: firstly, integration of evaluation strategies at the research study planning phase is likely to promote more effective community engagement. Secondly, a combination of quantitative and qualitative methods that draw on simple descriptive statistics and thematic analysis can provide nuanced perspectives about the value of community engagement. Thirdly, such evidence is necessary in establishing and promoting the science of community engagement in genomics research and health research more broadly.

## 1. Background

There is growing emphasis on community engagement as an essential component of promoting ethical best practice in global health research [1, 2]. In genomics research, genuine community engagement is increasingly advised (see, for example, [3, 4]) and is a requirement for the ethical use of broad consent in African genomics studies [5, 6]. Community engagement is a critical component of ethical standards for African genomics research, specifically in managing the potential risk of stigmatization [7]. It can also be used to foster conversations about ethical aspects of genomics research, including, for instance, conversations about the return of individual genetic research results [8]. Increased focus on the value of community engagement in research study planning, implementation, and reporting back of findings raises important questions about its ethical rationale [2, 9]. Equally important questions are then raised about the methods appropriate to achieving its objectives.

There is a small but growing body of work describing community engagement approaches in genomics both internationally (see, for example, [3, 10]) and more specifically on the African continent [11–15], but few studies describe or consider approaches to evaluating the effectiveness of the methods used. Those that do, such as O'Daniel et al. [3], typically draw from a quantitative pre-/posttest design, using questionnaires or surveys before and after community engagements to evaluate impact. As researchers in global health [16–19] and genomics research [20] grapple more with questions about how to determine the effectiveness of community engagement activities, one important challenge is emerging: prioritisation at the research study planning phase. Tindana et al. [20] highlighted that community engagement is often implemented as an afterthought to the design of the overall research project. Therefore, little, if any, time or resources go into its development, including setting clear goals and objectives, defining stakeholders, considering methods, and designing appropriate instruments. The result is that community engagement activities may be left to trial and error [20]. Newman et al. [17–19] and Tindana et al. [20] argued that one remedy would be to develop a science of community engagement. An essential component of this would be to develop evaluation strategies to assess the impact of community engagement methods on achieving stated objectives.

Through two African genomics studies, the Stigma in African Genomics Research study and the Genomics of Schizophrenia in South African Xhosa People (SAX) study, we identified a unique opportunity to contribute to building an evidence base for community engagement. These two studies were conveniently selected by the authors as they were genomics studies that the authors were research team members of at the time. This article reports on strategies used for evaluating community engagement that addressed the risk of stigma in these two studies. We compare the strengths and limitations of both community engagement strategies and outline ways in which they may inform future community engagement work in genomics research and health research more broadly.

## 2. Methods

### 2.1. Case 1: Stigma in African Genomics Research on Schizophrenia and Rheumatic Heart Disease Study

The Stigma in African Genomics Research on Schizophrenia and Rheumatic Heart Disease study explored the effect of genetic attribution on stigma relating to two disease groups: schizophrenia and rheumatic heart disease (RHD). The study was funded by the National Institutes of Health (Grant no. 1U01HG008226-01) and was a member of the H3Africa Consortium (http://www.h3africa.org). It was tethered to two ongoing genomics research projects involving patients with these conditions. Permission to implement and evaluate community engagement activities on this study was granted by the University of Cape Town, Health Sciences Research Ethics Committee (FHS204-2015).

#### 2.1.1. Aim of the Community Engagement Activity

Few platforms are available to South Africans with schizophrenia or RHD to openly discuss their illness and stigma experiences, yet such discussions may have an important influence on internalised stigma and perceived discrimination. Through community engagement, this study sought to target South Africans living with either schizophrenia or RHD, in the Western Cape Province, and engage them in a one-day experiential theatre workshop that allowed them to give voice to both their illness experiences and experiences of stigma. Participants were then contacted at two weeks and five months after the workshop to assess if and how participation in this community engagement activity had impacted on their self-reports of internalised stigma.

#### 2.1.2. The Target Populations

Working in collaboration with Fountain House, a Cape Town-based organization that provides psychosocial support aimed at empowering people with mental illness, we identified people from this organization with a diagnosis of schizophrenia. These individuals were utilizing the job placement and psychosocial support services offered by Fountain House as they transitioned from in-patient to outpatient care and recovery. Individuals needed to be outpatients, compliant with their medication, with no current psychotic symptoms, and able to give informed consent to participate in the workshop. Potential participants were invited to an information session about the workshop and asked to register for the event; complete an informed consent form that gave consent for the workshop to be video-recorded; and be available for follow-up for feedback about the day. Working in collaboration with the then ongoing Genetics of Rheumatic Heart Disease study team members at Groote Schuur Hospital, we invited people with RHD who had participated in the genomic study to participate in a similar workshop.

#### 2.1.3. The Workshop Process

Each workshop was led by a trained drama facilitator who used a combination of body work and photographs to engage participants in storytelling about their illness experiences. Participants were divided into smaller groups where they developed 5-minute plays that embodied a common illness experience shared by participants in that group. Resultant plays were performed to the larger audience and video-recorded. The audience was then asked to comment on what they had witnessed. Discussion often orientated around coping strategies that had been used in response to these painful and sometimes stigmatizing experiences.

#### 2.1.4. Plays Emerging from the Schizophrenia Workshop

Within the schizophrenia workshop, five short plays were developed. The first play focused on the topic of joining a Nongovernment Organization (NGO) such as Fountain House as an outpatient in recovery, highlighting the benefits and challenges of the recovery process. Some challenges included finding the right medication and remaining adherent despite the side effects, finding sustainable employment, and managing the stigma and discrimination that come from having the illness. Other challenges this group engaged with included learning to accept how one has changed as a result of their illness and the long-term consequences of living with a severe mental illness. The second play focused on the intersection of substance use and schizophrenia. Actors explored the painful conflicts that emerge in a home where family members are living with both severe mental illness and substance use, whether it be on the part of the person with schizophrenia or other family members. The third play highlighted the challenges of being a parent with schizophrenia, having limited employment prospects, but still having to manage the financial responsibilities of the family, especially the wants and needs of children. The fourth play was set in a psychiatric hospital and highlighted challenges people experienced when transitioning into the in-patient hospital setting for initial treatment. These challenges included patients having their daily routines restricted and feeling silenced and misunderstood by medical staff. The final play focused on the theme of negative thinking as a pervasive experience of people living with schizophrenia. Actors explored some of the negative messaging they contend with, such as “being a failure;” “being unable to survive;” “losing the ability to create and thrive;” and “the fear of not being able to recover.” Actors also explored how hope and positive thinking about recovery helped manage some of this negative thinking.

#### 2.1.5. Plays Emerging from the RHD Workshop

Within the RHD workshop, three short plays were developed. The first spoke about the process of undergoing heart surgery, the “fear of the uncertainty of the outcome,” and “anxiety about leaving home and going into the hospital space.” The other actors reflected emotions that family members grappled with including hopelessness and despair regarding fears of the negative outcome of the operation, and positive reactions of happiness when the operation was successful, and a renewed sense of hope for the future. The second play focused on how valuable time was and how fragile life was. Actors described the renewed sense of hope and faith that people living with RHD reported after a successful operation and the positive impact of this reaction on the recovery process and their overall quality of life. The third drama was centred on being in the hospital. It spoke of the many months some patients spend going in and out of theatre and hospital wards due to complications related to their valve replacement operation, resulting in heightened fear of death. Actors described how support mechanisms such as family, faith, and competent medical practitioners helped overcome this difficult process. Longer-term challenges faced by some patients after completing the operation were also portrayed, including limited opportunities to find employment and frequently accessing health services for medication.

Video extracts from each workshop were developed into DVDs. These were distributed to local psychiatric treatment facilities for showing to other patients recovering from schizophrenia and to RHD patients during patient awareness days. Each participant also received a copy of the DVD.

#### 2.1.6. Evaluation Strategy

The evaluation strategy for this community engagement activity included a combination of both quantitative and qualitative data, collected at three stages. In stage 1 at the start of the workshops, participants were asked to complete questions drawn from the Internalised Stigma of Mental Illness (ISMI) Scale [21, 22]. This provided a quantitative baseline measure of participants' internalised stigma experiences. Participants rated their own experiences of alienation and stigma resistance, in the form of 11 questions (outlined in Table 1). Questionnaire items about alienation assess experiences of being alienated or excluded from the society as a result of the person's illness [21]. Items about stigma resistance measure an individual's ability to resist or remain unaffected by stigmatizing experiences [21].

We expected that participants who experienced high levels of stigma would score high on the alienation questions. We anticipated that exploring illness and stigma experiences through the theatre workshops and hearing others' similar experiences and different ways of coping would challenge some of these stigmatizing beliefs and result in reduced endorsements of experiences of alienation and increased endorsements of stigma resistance over time.

In stage 2, participants were contacted via telephone, two weeks after the workshop, to complete these same 11 questions. In addition, participants provided qualitative feedback about their experiences of the workshops in response to four questions:

- Do you think that participating in the workshop has made you feel more open about talking about your illness experience, and if so, how?
- Has the information shared during the workshop made you think differently about how you cope with your illness, and in what ways?
- What do you think worked well during the workshop?
- What improvements would you suggest?

In stage 3, participants were contacted via telephone five months later and invited to video showing of the workshop. After showing, participants completed the same 11 questions and provided qualitative feedback to the following question:

- What, if anything, has changed for you since participating in this workshop?

Frequency of endorsements of the alienation and stigma resilience was compared at the time of the workshop (stage 1), two weeks later (stage 2), and five months later (stage 3) for both the schizophrenia and the RHD sample. The percentage of participants who indicated agree or agree strongly was calculated for each questionnaire item and then compared across the three steps. Qualitative responses were analysed thematically. All questions held equal weight in the analysis.

#### 2.1.7. Evaluation Results

A total of 25 people with schizophrenia and 20 people with RHD attended the theatrical workshops. Of these, 80% of participants provided feedback at stage 2 (schizophrenia workshop: *n* = 20; RHD workshop *n* = 16), and 45–50% provided feedback at stage 3 (schizophrenia workshop: *n* = 11; RHD workshop *n* = 10). The small sample size prevented us from running inferential statistics on the data, but instead, we explored the data descriptively using frequencies of item endorsements.

**Figure 1:**
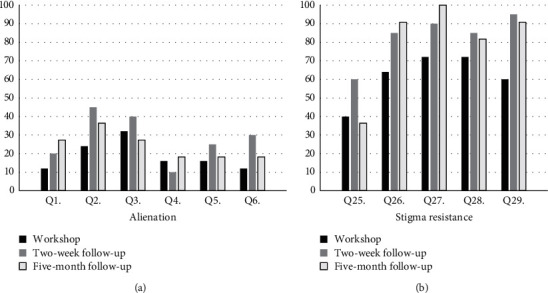
Schizophrenia workshop: % of endorsements of alienation and stigma resistance over time.

**Figure 2:**
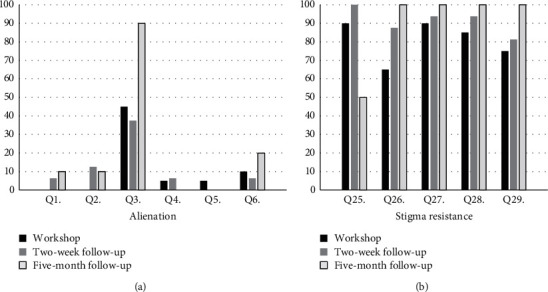
RHD workshop: % of endorsements of alienation and stigma resistance over time.

Overall, endorsements of alienation items were lower in the RHD group than the schizophrenia group and increased over time within both groups, particularly for question 2 (“Having schizophrenia has spoiled my life”) and question 3 (“People without schizophrenia couldn't possibly understand me”) in the schizophrenia group and question 3 in the RHD group. Endorsements of stigma resistance were higher in the RHD group in comparison with the schizophrenia group and increased over time for both groups, except question 25 (“I feel comfortable being seen in public with a person who obviously has schizophrenia/heart disease”). The percentage of participants who endorsed alienation and stigma resistance items at each of the 3 steps is presented in Figures 1 and 2.

There was considerable attrition of participants over time which likely inflated these findings, but they suggest that the unique illness experiences of living with schizophrenia and RHD were somehow amplified for participants, after engaging with the workshop and the resultant follow-ups. Qualitative feedback from participants about their workshop experience helped to contextualise this finding.

Qualitative feedback from both groups was generally very positive. At the two-week follow-up, participants from the schizophrenia workshop reported that they felt more understood, confident, and open to sharing their personal stories with those around them. Participants from the RHD workshop reported a sense of lightness, relief, and hope for the future after attending the workshop. Within this safe, accepting space, both groups' members were able to share painful lived experiences of the consequences and stigma they faced, reinforcing how alienating living with these illnesses can be at times.

Of the 20 schizophrenia group participants, 15 thought the workshop allowed them to be more open about their illness experiences, and 14 out of 16 RHD group participants felt the same way; 16 of the schizophrenia group and 11 of the RHD group thought the information shared during the workshop challenged how they coped with their illness. The most commonly reported strength of the workshop was the opportunity to engage with other people living with the same illness and listen to their lived experiences and ways of coping. In both groups, individuals shared how the group experience had motivated them to take a risk and engage differently in the environment around them. For one participant in the schizophrenia workshop, this meant applying for and securing employment, after hearing from his peers that others had full-time employment. For another participant in the RHD workshop, this manifested in her exploring fertility options, after hearing how other women in the group had successful pregnancies, and subsequently falling pregnant and delivering a healthy baby of her own. These are examples of two significant life changes that participants associated with their participation in these workshops.

At the five-month follow-up, participants watched the video recording of the workshop and were asked “What, if anything, has changed for you since participating in this workshop?” Participants from the schizophrenia group again reported feeling more confident in themselves, more positive, and motivated to participate in future tasks which allowed them spaces to share their illness experiences with others and greater acceptance for their illness. Some participants commented that knowing we were conducting this same workshop with people with heart disease reduced their own negative beliefs about schizophrenia. The RHD participants shared similar feelings of a reaffirmed sense of lightness, faith, and hope. Many participants also reported feeling stronger and having developed a better sense of self-confidence, awareness, and ability to be more open about their illness and educate others about RHD without as much fear of being rejected. These descriptions concur with the increasing endorsements of stigma resistance presented in Figures 1 and 2.

The feedback from both schizophrenia and RHD workshops suggests that these workshops aligned well with the original goals outlined for community engagement in that they created a space for participants to explore and openly discuss their illness and stigma experiences with others who shared the same illness. The nature of this experience appeared to impact positively in normalising some of the challenges of living with schizophrenia and RHD, even though participants became more aware of some painful stigmatizing experiences as they engaged in this process.

### 2.2. Case 2: The Genomics of Schizophrenia in South African Xhosa People (SAX) Study

The Genomics of Schizophrenia in South African Xhosa People (SAX) study aimed to identify genes or mutations underlying predisposition to schizophrenia in the South African Xhosa population. The study was funded by the National Institute of Mental Health (Grant no. 5U01MH096754) and was also part of the H3Africa Consortium (http://www.h3africa.org). Permission to implement and evaluate community engagement activities on this study was granted by the University of Cape Town, Health Sciences Research Ethics Committee (FHS049/2013).

#### 2.2.1. Aim of the Community Engagement Activity

During recruitment for the SAX study, which targeted Xhosa people with schizophrenia living in the Western and Eastern Cape Provinces, participants voiced a need for more education about severe mental illness and local psychosocial support structures that could assist in recovery. The SAX study team saw this as an opportunity to also address some of the mental health stigma surrounding schizophrenia within a local context. With this in mind, the team sought to develop Mental Health Literacy Day, which was conducted in isiXhosa and where all the speakers were Xhosa. Speakers included psychiatric nurses working as study recruiters on SAX, a community psychiatric nurse from Fort England Hospital, a local police officer, a local patient in recovery, and two mothers of sons living with schizophrenia. The aims of the community engagement were twofold: (1) create discussion about severe mental illness symptoms and treatment options and (2) create a better understanding of the lived experiences of people with schizophrenia and their families living in a particular area in the Eastern Cape of South Africa in order to challenge some of the mental health stigma about schizophrenia in the community.

#### 2.2.2. The Target Populations

Working in collaboration with Fort England Hospital, a psychiatric hospital in the town where we conducted the engagement, we identified approximately 100 Xhosa people with schizophrenia who were outpatients at the hospital, some of which had been recruited as participants in the SAX genomics study and their families. None of these had participated in the theatre workshops we described earlier. Potential participants were informed about the Mental Health Literacy Day and encouraged to attend. Transportation was arranged from collection points at three community psychiatric clinics to Fort England Hospital where the event was held.

#### 2.2.3. The Mental Health Literacy Day Programme

The day began with a welcome song performed by the Fort England in-patient choir. The hospital matron welcomed participants to the event, and six 10-minute presentations followed. Presentation topics included (1) an overview of the symptoms of severe mental illness and current medication and psychotherapy treatment options; (2) psychiatric services available within the community; (3) the complementary role that traditional healing may play alongside psychiatric care for people with severe mental illness; (4) the role of police in managing psychiatric emergencies and transitioning people to in-patient facilities; (5) a patient in recovery's perspective on living with schizophrenia; and (6) two mothers' perspectives on living alongside children with schizophrenia. The event concluded with a quiz session based on the presentations and a closing song from the choir before lunch was served. A local Xhosa newspaper article summarized the event and outlined psychosocial support services available in the community for Xhosa people with severe mental illness.

#### 2.2.4. Evaluation Strategy

The intention of the community engagement event was not only to address mental health stigma about schizophrenia, in a local context, by providing information that would improve the audience's knowledge about schizophrenia and related treatment options but also engage with the lived experiences of patients in recovery and their families. The evaluation strategy for this community engagement activity included a combination of both quantitative and qualitative data, collected at the end of the event.

1. Participants completed a rating of speakers on a three-point scale: “very helpful,” “not what I expected,” “very unhelpful.” To accommodate lower literacy levels, happy and sad facial expressions were used in conjunction with the scale descriptions. The scale was translated into isiXhosa.
2. Participants were also asked to provide a written response to one open-ended question, also translated into isiXhosa: what, if anything, has changed for you after attending this Mental Health Literacy Day?

**Figure 3:**
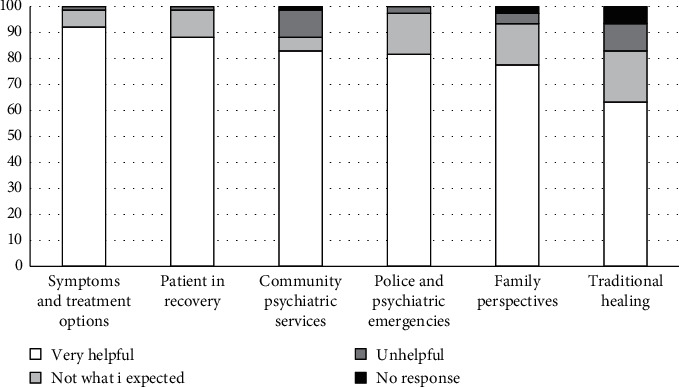
Mental Health Literacy Day presentation ratings.

The rating responses for each presentation are calculated as percentages and are summarized in Figure 3, while responses to the open-ended question are analysed for recurring themes. All questions held equal weight in the analysis.

#### 2.2.5. Evaluation Results

A total of 100 people attended the Mental Health Literacy Day. Of these, 76 completed the evaluation task. The “symptoms of schizophrenia and related treatment options” presentation was rated the most helpful on the day. This presentation was followed closely by the “patient in recovery” presentation. Presentations with the most unhelpful ratings included the “community psychiatric services” and the “traditional healing” talks. These results aligned with the two recurring themes which emerged in response to the open-ended question: “What, if anything, has changed for you after attending this Mental Health Literacy Day?” First, audience members reported being more knowledgeable about schizophrenia, its symptoms, treatment processes, and resources. Second, they commented that the day had challenged some of the negative views they themselves held about schizophrenia. This feedback suggested that the community engagement activity was well aligned with its original goals, namely, to create discussion about severe mental illness symptoms and treatment options and create a better understanding of the lived experiences of schizophrenia in order to challenge mental health stigma.

## 3. Discussion

In this paper, we described evaluation strategies for two community engagement events. The events aimed at managing stigma, conducted in the context of two African genomics research projects. Participants for these two events were diagnosed with either schizophrenia or rheumatic heart disease.

Our first example included a mixed-method evaluation strategy of rated and open-ended questions used to evaluate the impact of a one-day theatrical workshop. The workshop intended to give voice to the illness experiences of people living with schizophrenia and RHD. A particular strength of this evaluation strategy was being able to track changes in endorsements of alienation and stigma resistance over time and to understand these changes in the context of qualitative feedback from participants. However, this required careful planning and integration of the evaluation strategy into the community engagement activity at the start of the project and financial, time, and staffing resources in managing the evaluation process over time.

Our second example of community engagement evaluation involved a mixed-method strategy including a short rating scale and a qualitative response to a single open-ended question. The aim was to evaluate the impact of the Mental Health Literacy Day in addressing mental health stigma in the community. The appeal of this approach was its quick and easy implementation. The rating scale was easy to construct, and the open-ended question highlighted what was most impactful for participants on the day. One limitation was that the feedback was superficial and did not provide the team with insights into how to meaningfully improve this type of engagement in the future.

Three important learnings arose from these experiences. First, evaluation strategies become more meaningful when they are included as a component in the planning phase of community engagement activities. As such, these evaluation strategies should be integrated at the research study planning phase as a way of assessing the intended goals of community engagement activities [17–20]. Second, established assessment tools such as psychometric measures or even subscales of measures can be easily integrated into an evaluation strategy. The resultant data need not necessarily be used to run inferential statistics to determine significance. Rather, descriptive methods such as frequencies of item endorsements can help researchers understand how a community engagement activity is impacting on participants. One or two carefully considered open-ended questions such as “what, if anything, has changed for you after participating in this activity?” can be used to contextualise these results. The examples here illustrate how a mixed-method approach to evaluation is helpful in making deeper meaning of data typically drawn from quantitative pre-/postactivity evaluation strategies. Third, participation in community engagement activities appears to create a sense of momentum in some participants, which can have a powerful impact. One example in this study includes the man who participated in the schizophrenia workshop and applied for and secured employment after hearing of his peers' employments. Another example includes the woman from the RHD workshop who explored fertility treatment and consequently delivered a healthy baby after hearing of other women with RHD who are parents. It is only through mixed-method evaluation strategies that we were able to document evidence of this type of impact, reinforcing the valuable role community engagement plays in research contexts.

Both of these activities were meaningful approaches to community engagement in the context of genomics research because they played essential roles in enriching the research projects and in offering opportunities to respect the dignity and value of the participants involved in the project. For the Stigma in African Genomics Research study, the event opened a safe space where participants could give verbal and physical expressions to their illness and stigma experiences and learn from others about how they cope with their illness and feel empowered. Within the Genomics of Schizophrenia in South African Xhosa People (SAX) study, the Mental Health Literacy Day provided participants and their family members with an opportunity to learn more about the symptoms of schizophrenia, treatment options, and the lived experiences of patients in recovery and their families. Both events enriched the research team's understanding of the impact these two conditions have on the lives of people living with them. The events highlighted the challenges faced by the patients and their families and created personal relationships with some of the participants which formed a resource for further interaction and engagement. Understood as such, these community engagement events align with the approach to respect that King et al. proposed: such activities highlight and acknowledge elements of the research project that are important and valuable to the participants, as human beings [2].

## 4. Conclusions

In conclusion, these two case studies highlight the need for the integration of evaluation strategies at the research study planning phase to promote effective community engagement activities. Increased focus on the value of community engagement in research study planning, implementation, and reporting back of findings is key in establishing the science of community engagement. A combination of quantitative and qualitative methods that draw on simple descriptive statistics and thematic analysis can provide nuanced perspective into what participants find valuable about community engagement activities. Such evidence is necessary in establishing and promoting the science of community engagement in genomics research and health research more broadly.

## Figures and Tables

**Table 1:** Experiences of alienation and stigma resistance drawn from the Internalised Stigma of Mental Illness (ISMI) Scale.

*Alienation*
Q1. I feel out of place in the world because I have schizophrenia/a heart disease
Q2. Having schizophrenia/a heart disease has spoiled my life
Q3. People without schizophrenia/heart disease could not possibly understand me
Q4. I am embarrassed or ashamed that I have schizophrenia/a heart disease
Q5. I am disappointed in myself for having schizophrenia/a heart disease
Q6. I feel inferior to others who don't have schizophrenia/a heart disease

*Stigma Resistance*
Q25. I feel comfortable being seen in public with a person who obviously has schizophrenia/heart disease
Q26. In general, I am able to live life the way I want to
Q27. I can have a good, fulfilling life, despite my schizophrenia/heart disease
Q28. People with schizophrenia/heart disease make important contributions to society
Q29. Living with schizophrenia/heart disease has made me a tough survivor

## Data Availability

The data for each of these case study analyses used to support the findings of this study are included within the supplementary information file. These include the following: Case 1: C1: ISMI data—total item endorsements for alienation and stigma resistance collected during stages 1, 2, and 3; C1: Stage 2—qualitative responses received during stage 2 from both groups; C1: Stage 3—qualitative responses received during Stage 3 for both groups. Case 2: C2: Rating data—total ratings for the presentations.

## Abbreviations

ISMI: Internalised Stigma for Mental Illness scale
RHD: Rheumatic heart disease
SAX: Genomics of Schizophrenia in South African Xhosa People study.

## Supplementary Materials

Supplementary Materials Case 1: C1: ISMI data—total item endorsements for alienation and stigma resistance collected during stages 1, 2, and 3; C1: stage 2—qualitative responses received during stage 2 from both groups; C1: stage 3—qualitative responses received during stage 3 for both groups. Case 2: C2: rating data—total ratings for the presentations.
