# Supplementary material for: Evaluating Community Engagement Strategies to Manage Stigma in Two African Genomics Studies Involving People Living with Schizophrenia or Rheumatic Heart Disease
Source: Glob Health Epidemiol Genom. 2021 Jun 26;2021:9926495. doi: 10.1155/2021/9926495 (PMC8415068; doi:10.1155/2021/9926495)
Supplement: Supplementary Materials — Case 1: C1: ISMI data—total item endorsements for alienation and stigma resistance collected during stages 1, 2, and 3; C1: stage 2—qualitative responses received during stage 2 from both groups; C1: stage 3—qualitative responses received during stage 3 for both groups. Case 2: C2: rating data—total ratings for the presentations. [file 9926495.f1.zip › 9926495.f1/C1 ISMI Data.pdf]

	P1 S n=25	P1 % S n=25	P1 RHD n=20	P1 % RHD n=20	P2 S n=20	P2 % S n=20	P2 RHD n=16	P2 % RHD n=16	P3 S n=11	P3% S n=11	P3 RHD n=10	P3% RHD n=10
Alienation												
I feel out of place in the world because I have schizophrenia/a heart disease	3	12	0	0	4	20	1	6.25	3	27	1	10
Having schizophrenia/a heart disease has spoiled my life	6	24	0	0	9	45	2	12.5	4	36	1	10
People without schizophrenia/heart disease could not possibly understand me	8	32	9	45	8	40	6	37.5	3	27	9	90
I am embarrassed or ashamed that I have schizophrenia/a heart disease	4	16	1	5	2	10	1	6.25	2	18	0	0
I am disappointed in myself for having schizophrenia/a heart disease	4	16	1	5	5	25	0	0	2	18	0	0
I feel inferior to others who don’t have schizophrenia/a heart disease	3	12	2	10	6	30	1	6.25	2	18	2	20

Stigma Resistance

I feel comfortable being seen in public with a person who obviosuly has schizophrenia/heart disease	10	40	18	90	12	60	16	100	4	36	5	50
In general, I am able to live life the way I want to	16	64	13	65	17	85	14	87.5	10	91	10	100
I can have a good, fulfilling life, despite my schizophrenia/heart disease	18	72	18	90	18	90	15	93.75	11	100	10	100
People with schizophrenia/heart disease make important contributions to society	18	72	17	85	17	85	15	93.75	9	82	10	100
Living with schizophrenia/heart disease has made me a tough survivor	15	60	15	75	19	95	13	81.25	10	91	10	100
