# Supplementary material for: Evaluating Community Engagement Strategies to Manage Stigma in Two African Genomics Studies Involving People Living with Schizophrenia or Rheumatic Heart Disease
Source: Glob Health Epidemiol Genom. 2021 Jun 26;2021:9926495. doi: 10.1155/2021/9926495 (PMC8415068; doi:10.1155/2021/9926495)
Supplement: Supplementary Materials — Case 1: C1: ISMI data—total item endorsements for alienation and stigma resistance collected during stages 1, 2, and 3; C1: stage 2—qualitative responses received during stage 2 from both groups; C1: stage 3—qualitative responses received during stage 3 for both groups. Case 2: C2: rating data—total ratings for the presentations. [file 9926495.f1.zip › 9926495.f1/C1 Stage 2 Qualitative.pdf]

| RHD | Q1 | Explain | Q2 | Explain | Q3 | Q4 |
|---|---|---|---|---|---|---|
| 1 | yes | It made him feel much better. He enjoyed being with the people. | yes | Made me more open about my illness | the plays | the walk up the hill |
| 2 | yes | The workshop counselled he. She feels relieved | yes | Made me see people have different stories, different ways of coping. Gave me strength and motivation to stay positive | being with other patients | no |
| 3 | yes | He didn’t hide it. The workshop provided a space to encourage others | No | Made me more positive | beng with people who understood me | make it longer |

| | | | | | | |
|---|---|---|---|---|---|---|
| 4 | yes | Its not shameful to have a RhD. She is open about it. Felt excited to be part of it and helping others | No | | the plays | no |
| 5 | yes | He was scared at first but got better over time | yes | other group comments made him realise how to deal with different issues he hadn't experienced yet | yes comments from the group | no |
| 6 | yes | She felt light after the workshop | yes | gave her other ideas about what to do, when, how to take care of herself. Helped her know she is not alone | the plays | more support for patients, larger group |

| | | | | | | |
|---|---|---|---|---|---|---|
| 7 | yes | She felt relieved | yes | She felt free, she saw others with her illness and pace maker and now she can tell anybody | the plays - similar to her story | no |
| 8 | yes | She learnt new things | yes | made her more comfortable with herself and happy | the plays - taught her about her and others' experiences | do another workshop - something different |
| 9 | yes | She felt excited to be there and share her experiences | yes | it was a relief knowing how people were cared for by the doctors and hospital. She felt able to share her stiry because others shared theirs | being with other patients. Being with people who understood me | no |

| | | | | | | |
|---|---|---|---|---|---|---|
| 10 | yes | people were anxious to start but felt more relaxed as the day continued. It made everyone more open | no | | attention of facilitators | no |
| 11 | yes | She felt more hopeful. There are others her own age who have the illness. She isn't the only one. She could maybe also have a child | yes | | | no |
| 12 | yes | It made her right | yes | giving and getting different adviced helped her to think differently | the plays | no |

| | | | | | | |
|---|---|---|---|---|---|---|
| 13 | yes | Felt free to share after hearing others | yes | hearing other peoples' stories | the plays | no |
| 14 | yes | people were anxious to start but felt more relaxed as the day continued. It made everyone more open | yes | it helped to hear other peoples' stories and how many positive stories there were | taking away advice on how to handle challenges | invite more people, spread awareness, target the public |
| 15 | | No responses | | | | |
| 16 | | No responses | | | | |

SCH

| | | | | | | |
|---|---|---|---|---|---|---|
| 1 | yes | The workshop made him feel ok about sharing his feelings with others | yes | Made him think differently about people and how they shared their experiences | the plays | no |
| 2 | no | | no | He copes well with his illness. He isnt shy to say he has schizophrenia | the plays | no |
| 3 | yes | It relieved his stress. He feels better about talking about his illness | yes | He felt more hopeful | open discussion led by patient stories | no |

| | | | | | | |
|---|---|---|---|---|---|---|
| 4 | yes | Never been in a community of people with schizophrenia before. Made him more comfortable to talk in a group and share his experiences | yes | Learnt it is important to drink your pills religiously | group work with other patients | no |
| 5 | yes | informative, gave her tips on how to cope better | yes | Exposed her to other ways of coping she hadn't thought of | people opening up and sharing their experiences and feelings | need for ongoing workshops |
| 6 | yes | speaking to other patients made her feel more comfortable sharing her own experiences with others | yes | reminded her that people don’t always understand her at times, and she mustn't get upset by that | the plays and workshop as full day event | for those not wanting to be recorded to have the opportunity to participate anyway |

| | | | | | | |
|---|---|---|---|---|---|---|
| 7 | yes | made her feel open and able to talk about anything and express her character | yes | It made her open up and share how she copes with the group | the plays | no |
| 8 | yes | helped her to feel more open by having a platform to speak and share | no | | people opening up and sharing their experiences and feelings | no |
| 9 | yes | speaking to other patients made her feel more comfortable sharing her own experiences with others. She felt very understood | yes | helped her to get tips on how to cope with side effects from the medication | seeing the change in the group from negative to positive | more workshops more frequently |

| | | | | | | |
|---|---|---|---|---|---|---|
| 10 | yes | speaking to other patients made her feel more comfortable sharing her own experiences with others. She felt very understood | yes | other peoples' stories helped him learn more about his illness and different ways of coping with it | patients connecting with one another | No |
| 11 | yes | gained confidence, and sourced a job | yes | gave him a new perspective. No longer feels sorry for homself and believes he can contribute to society and his family | Motivation from fellow patients on how to grow and be stronger | Topic for new workshop: watching video and discussing how it relates to people with schizophrenia |
| 12 | yes | made him feel more confident to speak about his illness to other people | yes | was helpful to hear how others dealt with their illness | being with other patients and sharing information | making the workshop more accessable to working people - after hours |

| | | | | | | |
|---|---|---|---|---|---|---|
| 13 | No | | Yes | Feels more positive when confronting challenges | Good to be around other patients | Adding arts and crafts to the programme like painting, clay etc |
| 14 | Yes | Very helpful to be able to share experiences with the group | Yes | It was very informative. She learnt about how others manage similar challenges | Very well organised | No |
| 15 | no | | No | | talking about his illness | no |

| | | | | | | |
|---|---|---|---|---|---|---|
| 16 | no | found it difficult to concentrate in the group because she had forgotten her medication | yes | made her think of past experiences she hadn't spoken about for a while, and the group support helped her talk about them | the plays | hot inside the hall |
| 17 | yes | first time sharing in a group with other patients who had similar experiences | yes | Fun and entertaining so it brought a lighter side to talking about his illness | using drama a different approach | no |
| 18 | yes | it inspired her | yes | She shared her experiences and this made her open up more | the plays | no |

| | | | | | | |
|---|---|---|---|---|---|---|
| 19 | yes | he learnt a lot from shared experiences of others and enjoyed the interaction of the workshop | yes | Made him realise its not only him. The group shared about different ways of coping | the plays | no |
| 20 | | No responses | | | | |
