# Supplementary material for: Evaluating Community Engagement Strategies to Manage Stigma in Two African Genomics Studies Involving People Living with Schizophrenia or Rheumatic Heart Disease
Source: Glob Health Epidemiol Genom. 2021 Jun 26;2021:9926495. doi: 10.1155/2021/9926495 (PMC8415068; doi:10.1155/2021/9926495)
Supplement: Supplementary Materials — Case 1: C1: ISMI data—total item endorsements for alienation and stigma resistance collected during stages 1, 2, and 3; C1: stage 2—qualitative responses received during stage 2 from both groups; C1: stage 3—qualitative responses received during stage 3 for both groups. Case 2: C2: rating data—total ratings for the presentations. [file 9926495.f1.zip › 9926495.f1/C1 Stage 3 Qualitative.pdf]

RHD Group discussion

It looks like we had fun... Fun, fun…

Has anything significant changed for you after the workshop?
Yes, being involved in the workshop helped me a lot. I felt like I am not the only one with this disease. My life has changed since that day, I now tell myself that I need to accept my illness and accept myself the way that I am.

Yes I really really enjoyed that day. That was the best day every. I felt so free, I felt lighter. After that I was stronger, even though I got sick with small things that got me in hospital, but I felt like I am strong and I can overcome those things.

Any comments or feedback after you have watched yourselves performing and remebering that day.

I would like to greet everyone, and acknowledge that God has kept us until we could meet again today. Uh, I would say, after watching the videos, for me I would like to say, nothing is as important as hope and faith. Because we did not know.. And I am grateful to the doctors and their helpers for giving us this opportunity. Because in my life, I never thought that I would act and perform and then watch myself appear on a screen. But because of their strength from God, I was able to do that. I would also like to thank God to for giving us a brain to think. Also for the strength of the doctors who are able to think and help people in the country to heal and live again. I never thought that I could be one with others and act in a film, I am very grateful for that.

I would like to greet everyone. In this video and after that day, I realised a particular character about myself. I realised that I am strong. I can help other people and be a source of inspiration to them. Especially those who are for example scared to do the operation, and those living with RHD. From this drama I realise how important it is to do the research, and how it can relate to so many people with RHD. As many people are scared to face the illness, and for me it was not easy, but God gives us the strength to support one another. One thing that I got from that day is a sense of hope, we gave one another hope.

Why are others so quiet? You don't believe that you saw yourself on the screen.

Do others perhaps agree?
Group says yesss..

God has love. He gave us strength, one thing I noticed is that before I went there I was scared to talk about my illness in front of others. After we came to the workshop it gave us a space to talk to one another about our experiences and we realised that we are not alone. I realised that other people from different cultural and racial groups also experience some of the things which I experience. That is why I am saying. I thank God all the time for the opportunity.

Hello everyone. For me, you know arriving there that morning, I am sure everyone was in anticipation, maybe even nervous. Can I do this, can we do that, never done it before. Uhm, you know lots of people never had the platform to do that. I think it was remarkable how it all enfolded. Everyone assumed their roles which thay were placed in. And I am sure to their astonishment people were amazed and felt, did I really do that? Because it was out of character or league to perform. With that I think it opened up that dark side about fear of RHD, and I think maybe because of the window that opened up we realised we were not alone in this whole thing. Well done to all of us. Most of all to people doing the research, its excellent for tomorrow, what you have established may perhaps eradicate RHD and keep going.

I learnt that I need to have hope and believe. In God for example, if you have faith your things will work out well even in the operation process. You need to keep the faith that we established that day, that is what I try to do.

We all come from different walks of life but we met, and talked about the same thing and we became one. I think its like everyone said, hope, faith, development.

Enjoyed the day, but nothing has changed.

I realised on that day that my dream of having a baby can come true. Many women at the workshop shared their stories of how they have had children after the diagnosis. I am hopeful now that I can have a child of my own. I got a contact for the fertility clinic and therefore I set up an appointment, we are busy with the process to try and help me have a baby.

I couldn't believe what I did that day. I was so confident. I was happy to be there. Now I know I can do many things, even those I didn't think I would ever do. I am also more confident to talk about my illness.

So after sharing the personal stories and looking through the video, is there anything that this makes you think of
It reminds me of my 2 and half months period in hospital, after the operation. Going home and the long journey, but resulted in success.

It reminds me of my past, it shows me that I need to always be ready to cope with anything that may come your way. But it's a long journey.

You must just have hope, because after hope and faith comes death. So from these stories we can see that we need to have hope. God sends people, and I believe that he sent people in this group, doctors, and nurses as they help us in our lives. The health staff need to continue to encourage, acknowledge and researching or doing work around RHD.

So how was it like to see yourself on film?
I don’t believe that that was me. Yho. Hai, I cant believe it. Its amazing.

I have been waiting for the moment to see myself on this video. I have been excited. Everytime I come to the hospital, or like when we had the other RHD event in Cape Town in September I was hoping I get to see myself in the video. I am so grateful and glad that you and the doctors have done this for us. It means so much to me. My family all watched the video and they are so proud of me for having the confidence to share my experience on that day. My family shows that video even to people who comes to visit at home.

I am happy to see myself.

I was very happy to see myself, even [Mzabantu Vusani] my husband. I showed him the video, he was also happy to see both of us there in the group. It was a good day. Thank you.

Schizophrenia Group discussion

M: Has anything significant changed for you after the workshop?
Yes, it has actually made me realise that I can still live a normal life. I can actually look for work. Even though its not easy to find work when you have a mental illness, but I am still looking for a job at the moment. I am praying that I get one.

M: Have you been more motivated to find work?
More motivated yes.

I'm not bragging or anything hey, but I think the diagnosis that I have (is schizoafftective) and now I learnt, I have fun with myself. You know what I mean, like I have fun with people and interacting with people. You know what I am saying, I like being schizoaffective. Instead of being negative about my illness I am more positive about it. I have with myself, I have fun with people and interacting with people. Its just I like being schizoaffective.

M: So you are saying there is something about coming to terms with your illness which has allowed you to see things differently?
To see life differently yes, because like I am saying because if you have schizophrenia you have a personality disorder. But I don’t know, schizoaffective is different from schizophrenia, I don’t know why but I am finding it to be at peace. I can deal with people, even if they dont know who I am. I know who I am. ANd they can say what they want to. Because, like the stigma, its not their business why. It is my business and my families business and my chosen friends to know who I am.

M: Has that changed after coming to the workshop in some way?
Yes, uhm it doesn't make me feel ashamed that I am like this. It made me feel proud of who I am and how far I came in my life.

The illness helped me realise that we are all the same people. The other one is diabetic, the other one maybe has heart failure and other one has heart disease. So we are all the same as people. There is no difference between someone who has schizophrenia and a person who has got a diabetes or high blood pressure. I have learnt also that there are people who have both high blood pressure and schizophrenia. And I only have schizophrenia.

M: So you are saying that there is something that has shifted for you about schizophrenia, its an illness like other illnesses out there and you can use medication and manage it?

The doctor always says, its all about your maintaince. Its your brain your heart and your soul. In order to go forward. You mustnt be weak, you must strengthen yourself.

M: So you must find ways to cope?
Yes

M: Do you think there was something about that in participating in the workshop?

Yes, because when were participating in the workshop, you become active. You don’t become passive. You have to participant and by participating you satisfy yourself and become more positive in your life. Like before the play, you come to relax yourself, I dont know how to say..

M: Do other people feel that participating in the workshop allowed you to feel more comfortable in yourself and in your skin?
It made me feel more comfortable because we all had schizophrenia. And like talking to somebody that understand what you going through.

My life hasn’t really changed, but I am in a good space.

M: You say you are in a good space before?
I just am.

Not really anything changed. But to be motivated, you need to come to FH everyday, and everyday is a different story. By participating in the workshop, you are doing the work. Making it easy and trying to get over your illness. You become a stronger person. So people recognise you and make you feel as a warm person because you have the same disease. You feel comfortable around people around you. There is an acceptance. Its a consistent working thing, so we always doing something new at FH.

M: What changed for you after watching the video?
Nothing really, it was a bad picture of me.

One person when I was in Lentegeur, she was talking about your tablets and food and how it affects you. She was talking about how coffee can make you sleep and can affect your moods stuff like that. But exercise, walking progress in mind is good.

M: What you saying is that sharing of coping strategies, of what works well and what doesn't, is this helpful?
Yes, like now you know how you can get better.

Hopefully the programme will help others, because we had a schizophrenia workshop last week and some people who were at the workshop went there. So hopefully it will help them with their problems.

I had like a slight relapse after it. My doctor just said it might be the intensity of the workshop. It was something new and different. But I enjoyed it, I stuck it out and I enjoyed it. It was nice to be with everybody, but I felt at ease. It was difficult, but I enjoyed it.

What prompted you to follow with your doctor?

Uhm I see my doctor once a month, I didn't go specifically to see her after the workshop because it was over in like two days. But when I told her she said it's the anxiety of change, and doing something big.

M: So that was a big challenge for you to come and participate?

Yes, because I have always been by myself. Except perhaps at school. But even at school I had trauma, but even at school it was difficult for me.

M: Would you do it again?
Yes I would.

M: Do you think that being at that workshop would equip you better?
Ya, it might. That would be interesting to know, lets see. Because I have had the experience.

M: Did anybody else have difficulty after participating in the workshop?

Remember when you said that if you don't want to be in front of the camera you don't have to. So for me it was abit stressful when I saw that someone is recording. But its okay, so long as it helps someone. For me it was like I was a movie star and people are like all over me.

It is quiet a sureal experience to see yourself on screen.
Its nice to have a movie from FH by FH. Thank you very much.

Uhm, it was comforting being around people with the same illness, uhm I wasn't nervous or anything, I was just at ease. But uhm, I know what everyone is going to say, I laugh or watever, but I don't, I am obsessed about my weight. Its so embarrassing for me. I don't like seeing myself on screen. But i laught. That was a bit difficult for me, because they were photographing me, or videorising me.

Its made me realise, that its important to realise that we are all in the same boat. Its important to talk about it. I remember that Justine was saying that she is in a really bad space after the workshop, I talked to her and she said thanks, you know.
And I did feel better.

It made me realise that the way I was talking. It made me ask like what my doctor would ask me, and also my family. My mother she sees to it that I take my medication everyday at the same time. It ca therefore also let other people know that you must take your medication everyday at the same time.

So that's interesting, because on the one hand its difficult to have all these people telling you what to do and caring for you, but on the other hand people are giving me this information because they care about me. So there is a conflict there. So its hard.
Yes, it is hard. Like Yolande was saying, you must take your medication everyday at the same time. Sometimes its hard. I tend to take my medication late, but last year I was in church, I just had a back out. Then I realised that I need to take my medication at the same time everyday. And once my mother was very cross with me on that day, but I told my mother I have to do this.

It was awesome. Nothing changed but I felt like a superstar.

Maybe another play at FH.

Nothing has changed. Its still the same. It was good. He says he would do it again.
