# Supplementary material for: Evaluating Community Engagement Strategies to Manage Stigma in Two African Genomics Studies Involving People Living with Schizophrenia or Rheumatic Heart Disease
Source: Glob Health Epidemiol Genom. 2021 Jun 26;2021:9926495. doi: 10.1155/2021/9926495 (PMC8415068; doi:10.1155/2021/9926495)

	Very helpfı	Not what I	Unhelpful	No response
Symptoms and treatment options	92.10526	6.578947	1.315789	0
Patient in recover	88.15789	10.52632	1.315789	0
Community Psychiatric services	82.89474	5.263158	10.52632	1.315789
Police and psychiatric emergencies	81.57895	15.78947	2.631579	0
Family perspectives	77.63158	15.78947	3.947368	2.631579
Traditional healing	63.15789	19.73684	10.52632	6.578947

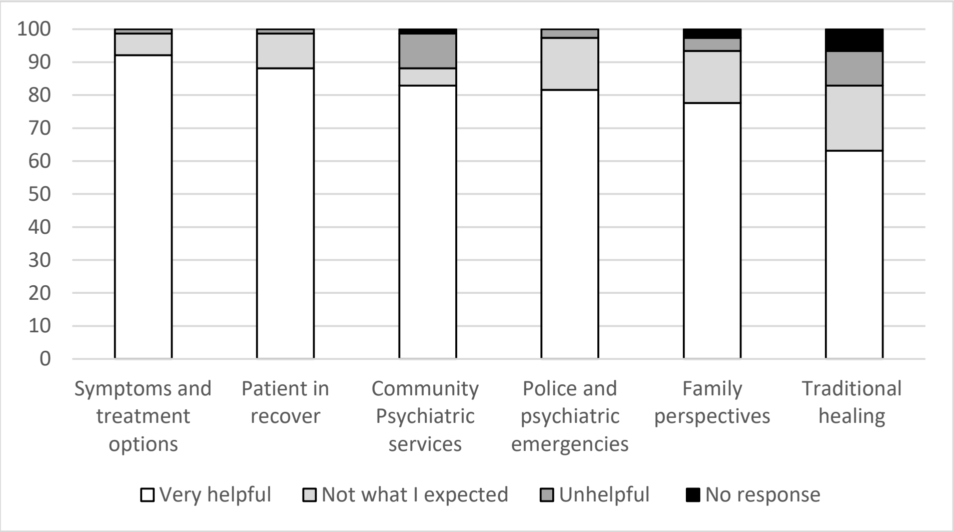

Supplement: Supplementary Materials — Case 1: C1: ISMI data—total item endorsements for alienation and stigma resistance collected during stages 1, 2, and 3; C1: stage 2—qualitative responses received during stage 2 from both groups; C1: stage 3—qualitative responses received during stage 3 for both groups. Case 2: C2: rating data—total ratings for the presentations. [file 9926495.f1.zip › 9926495.f1/C2 Rating Data.pdf]
