# Supplementary material for: Evaluating Community Engagement Strategies to Manage Stigma in Two African Genomics Studies Involving People Living with Schizophrenia or Rheumatic Heart Disease
Source: Glob Health Epidemiol Genom. 2021 Jun 26;2021:9926495. doi: 10.1155/2021/9926495 (PMC8415068; doi:10.1155/2021/9926495)
Supplement: Supplementary Materials — Case 1: C1: ISMI data—total item endorsements for alienation and stigma resistance collected during stages 1, 2, and 3; C1: stage 2—qualitative responses received during stage 2 from both groups; C1: stage 3—qualitative responses received during stage 3 for both groups. Case 2: C2: rating data—total ratings for the presentations. [file 9926495.f1.zip › 9926495.f1/SUPPLEMENTARY DESCRIPTION.docx]

**SUPPLEMENTARY DESCRIPTION**

These include: Case 1: C1 ISMI data - total item endorsements for alienation and stigma resistance collected during stages 1,2 and 3; C1 Stage 2 - qualitative responses received during stage 2 from both groups; C1 Stage 3 - qualitative responses received during stage 3 for both groups. Case 2: C2: Rating Data - total ratings for the presentations.
